# Anterior Urethral Strictures in Children: Disease Etiology and Comparative Effectiveness of Endoscopic Treatment vs. Open Surgical Reconstruction

**DOI:** 10.3389/fped.2019.00005

**Published:** 2019-01-31

**Authors:** Malte W. Vetterlein, Lars Weisbach, Silke Riechardt, Margit Fisch

**Affiliations:** Department of Urology, University Medical Center Hamburg-Eppendorf, Hamburg, Germany

**Keywords:** endoscopy, iatrogenic disease, mouth mucosa, pediatrics, urethral stricture

## Abstract

Pediatric anterior urethral strictures are rare and recommendations regarding treatment strategies derive from small monocentric case series. In 2014, a collaborative effort of the Société Internationale d'Urologie and the International Consultation on Urological Diseases drafted the first systematic and evidence-based guideline for diagnosis and treatment of urethral strictures in children. Against this backdrop, we performed an updated literature review to provide a comprehensive summary of the available evidence and contemporary outcomes with a focus on comparative effectiveness of endoscopic treatment (dilation or urethrotomy) vs. open surgical reconstruction. Overall, 22 articles reporting on children with anterior urethral strictures were included into the review. Most strictures were iatrogenic (48%) and traumatic (34%), whereas congenital (13%), inflammatory (4%), or postinfectious strictures (1%) were rather rare. The cumulative success rate of endoscopic treatment and urethroplasty was 46% (range: 21–75; *N* = 334) and 84% (range: 25–100; *N* = 347), respectively. After stratifying patients according to urethroplasty technique, success rates were 82% (range: 25–100; *N* = 206) for excision and primary anastomosis, 94% (range: 75–100; *N* = 40) for graft augmentation, 97% (range: 87–100; *N* = 30) for flap urethroplasty, and 70% (one study; *N* = 20) for pull-through urethroplasty. In conclusion, endoscopic approaches are rather ineffective in the long-term and open surgical reconstruction via urethroplasty should be preferred to avoid multiple, repetitive interventions. Future research may involve multi-institutional, collaborative, and prospective studies, incorporating well-defined outcome criteria and assessing objective surgical endpoints as well as patient-reported functional outcomes.

## Introduction

Anatomic lower urinary obstruction is a rather rare problem in pediatric urology. Whereas, voiding difficulties are commonly diagnosed in boys with urethral valves or hypospadias, the experience in treatment of isolated pediatric anterior urethral strictures is remarkably scarce. Thus, information on pediatric or adolescent strictures is commonly extrapolated from the adult literature (1). With exception of the collaborative 2014 Société Internationale d'Urologie (SIU) and International Consultation on Urological Diseases (ICUD) task force to generate evidence-based recommendations for those patients (2), there are literally no keystones to base evidence-based treatment considerations on. Given that urethral stricture disease is relatively rare in adults (3), the incidence in children is even smaller and there is a paucity of experience in patients up to 18 years. As a consequence, etiology and management in pediatric urethral stricture disease are not well defined. In this review, we aimed to provide a contemporary overview of available operative techniques, treatment approaches, and outcomes in this subgroup of patients.

## Materials and Methods

We performed a literature review through PubMed for articles published between 2010 and September 2018 on urethral strictures in children using the search term “urethr^*^ AND (child^*^ OR ped^*^) AND stricture.” Additionally, we included select evidence from the recent landmark reviews by Kaplan et al. (1, 2). We excluded articles, which reported on adult patients only, isolated posterior strictures given the entirely different etiology in most cases, or those without information on (standardized) outcomes. Of note, we also considered articles in which patients with strictures of the anterior urethra were pooled together with children suffering from posterior urethral strictures. Articles about re-operative surgery after previously failed hypospadias repair were excluded if neo-urethral strictures were not mentioned and described in detail, given that re-do hypospadias repairs may not generally implicate urethral stricture disease. We tabulated information on authors and year of each study, number of included patients, stricture etiology, location, and length, the surgical approach, age at surgery, the chosen definition of treatment success, success rates, length of follow-up, and the type of study design. Of note, we chose to summarize conservative success rates after the very initial treatment. We believe this is important to mention, given that some authors do report “overall” success rates derived from several subsequent treatment sessions (e.g., after multiple urethrotomies).

After applying selection criteria, 22 articles reporting data on 682 patients were included into analyses. Treatment approaches varied across the studies with seven articles reporting on urethrotomy or dilation (4–10), 11 articles reporting on urethroplasty (11–21), and four articles reporting on both techniques in comparative analyses (22–25). A summary of all articles considered for this review is depicted in Tables 1–3.

**Table 1:** Summary characteristics of included studies of patients who underwent urethroplasty for pediatric urethral stricture.

**Author**	**Year of study**	**Number of patients**	**Stricture etiology**	**Stricture location**	**Stricture length**	**Surgical approach**	**Age at surgery**	**Definition of treatment success**	**Success rate**	**Length of follow-up**	**Type of study**
Ashraf et al. (12)	2018	5	Inflammatory (*N* = 5)	Distal/Penile (*N* = 5)	Median 2.7 cm; Range: 1.5–4.0	One-stage Asopa BMGU (*N* = 5)	Median: 14 years; Range: 11–18	Functional success (i.e., improvement in uroflowmetry, patient satisfied with urinary stream)	100%	Median: 34 months; Range: 30–42	Retrospective
Aldaqadossi et al. (11)	2018	23	Iatrogenic (*N* = 16); Postinfectious (*N* = 4); Congenital/Unknown (*N* = 3)	Bulbar (*N* = 15); Bulbopenile (*N* = 8)	Mean: 5.1 ± 1.0 cm	Circular penile fasciocutaneous flap (*N* = 23)	Mean: 9.3 ± 2.6 years	Normal voiding, no radiographic manifestation of stricture, no further postoperative interventions	87%	Mean: 52 ± 17 months	Retrospective
Djordjevic et al. (13)	2011	15	Iatrogenic (*N* = 15)	Penile (*N* = 15)	Mean: 2.6 cm; Range: 3.0–5.5	One-stage ventral BMGU with tissue coverage and graft hanging (*N* = 15)	Mean: 13 years; Range: 9–17	Adequate urethral diameter, confirmed by uroflowmetry and urethrography	100%	Mean: 37 months; Range: 17–73	Retrospective
Hafez et al. (14)	2005	35	Traumatic (*N* = 35)	Bulbar (*N* = 9); Bulbopenile (*N* = 2); Posterior (*N* = 24)	Mean: 2.6 cm; Range: 1–5	EPA (*N* = 35)	Mean: 11.9 ± 3.4 years; Range: 6–18	Asymptomatic voiding without clinical evidence of residual stricture (good flow rate and absence of residual urine)	89%	Mean: 46 months; Range: 6–132	Retrospective
Pfalzgraf et al. (15)	2012	17	Traumatic (*N* = 8); Iatrogenic (*N* = 7); Congenital/Unknown (*N* = 2)	Bulbar (*N* = 13); Penile (*N* = 1); Posterior (*N* = 3)	Mean: 2.5 cm; Range: 0.5–4.0	EPA (*N* = 8); One-stage BMGU (*N* = 7); Staged BMGU (*N* = 2)	Median: 9 years; Range: 1–13	No subsequent procedure required	89%	Median: 30 months; Range: 4–115	Retrospective
Rourke et al. (16)	2003	17	Traumatic (*N* = 12); Iatrogenic (*N* = 3); Congenital/Unknown (*N* = 2)	Bulbar (*N* = 8); Posterior (*N* = 8); Bulbopenile (*N* = 1)	Median: 2.2 cm; Range: 1.0–6.0	EPA (*N* = 14); Penile flap augmentation (*N* = 2); One-stage BMGU (*N* = 1)	Median: 13.4 years; Range: 7–17	Urinary continence with a patent urethra and absent lower urinary tract symptoms	94%	Median: 57 months; Range: 12–124	Retrospective
Shenfeld et al. (17)	2008	14	Traumatic (*N* = 9); Iatrogenic (*N* = 1); Congenital/Unknown (*N* = 4)	Bulbar (*N* = 7); Posterior (*N* = 7)	N/S	EPA (*N* = 11); One-stage BMGU (*N* = 2); Penile flap augmentation (*N* = 1)	Children (*N* = 5)—Mean: 10.8 years; Range: 9–13; Adolescents (*N* = 9)—Mean: 16.7 years; Range: 14–18	No subsequent procedure required	93%	Mean: 30 months; Range: 12–54	Retrospective
Sunay et al. (18)	2011	75	Iatrogenic (*N* = 5); Traumatic (*N* = 70)	Bulbar (*N* = 38); Posterior (*N* = 37)	Mean: 2.3 cm; Range: 1.5–5.0	EPA (*N* = 54); Pull-through urethroplasty (*N* = 20); Ureteral graft augmentation (*N* = 1)	Mean: 12.3 years; Range: 6–17	Urinary continence, patent urethra, and absence of lower urinary tract symptoms	EPA: 69%; Pull-through urethroplasty: 70%; Ureteral graft augmentation: 100%; Overall: 69%	Mean: 43 months; Range: 12–94	Retrospective
Trachta et al. (19)	2016	8	Traumatic (*N* = 8)	Bulbar (*N* = 4); Posterior (*N* = 4)	N/S	EPA (*N* = 8)	Mean: 12.3 years; Range: 5–17	No evidence of stricture in urethrography	25%	Mean: 4.5 years; Range: 0.5–10	Retrospective
Vashishtha et al. (20)	2014	52	Traumatic (*N* = 30); Inflammatory (*N* = 20); Congenital/Unknown (*N* = 2)	Bulbar and Posterior; N/S	N/S	EPA (*N* = 52)	Mean: 10.8 years; Range: 4–18	N/S	81%	Mean: 42.8 months; Range: 12–144	Retrospective
Voelzke et al. (21)	2012	26	Traumatic (*N* = 26)	Anterior (*N* = 8); Posterior (*N* = 18)	Anterior strictures—Median: 2.5 cm; Range: 1.5–5.5; Posterior strictures—Median: 2.0 cm; Range: 1.0–5.0	One-stage BMGU (*N* = 3); EPA (*N* = 23)	Mean: 15 years; Range: 4–18	No evidence of stricture in urethrography, sufficient uroflowmetry, absence of lower urinary tract symptoms	Anterior strictures: 88%; Posterior strictures: 89%; Overall: 88%	Median: 0.7 years; Range: 0.07–7.8	Retrospective

*BMGU, buccal mucosal graft urethroplasty; EPA, excision and primary anastomosis; N/S, not specified*.

## Stricture Etiology and Location

According to the SIU/ICUD consultation on urethral stricture nomenclature (26), stricture etiology should be stratified into iatrogenic (e.g., hypospadias-associated, post-catheterization, etc.), traumatic, inflammatory (e.g., lichen sclerosus-associated), postinfectious, and congenital. Emphasis should be put on the term “congenital stricture,” which is a less common subcategory, and the diagnosis should generally only be made in the absence of urethral manipulation, infection, inflammation or trauma (26). Accordingly, patients without evident etiology were classified as “congenital/unknown” within this review.

**Figure 1:**
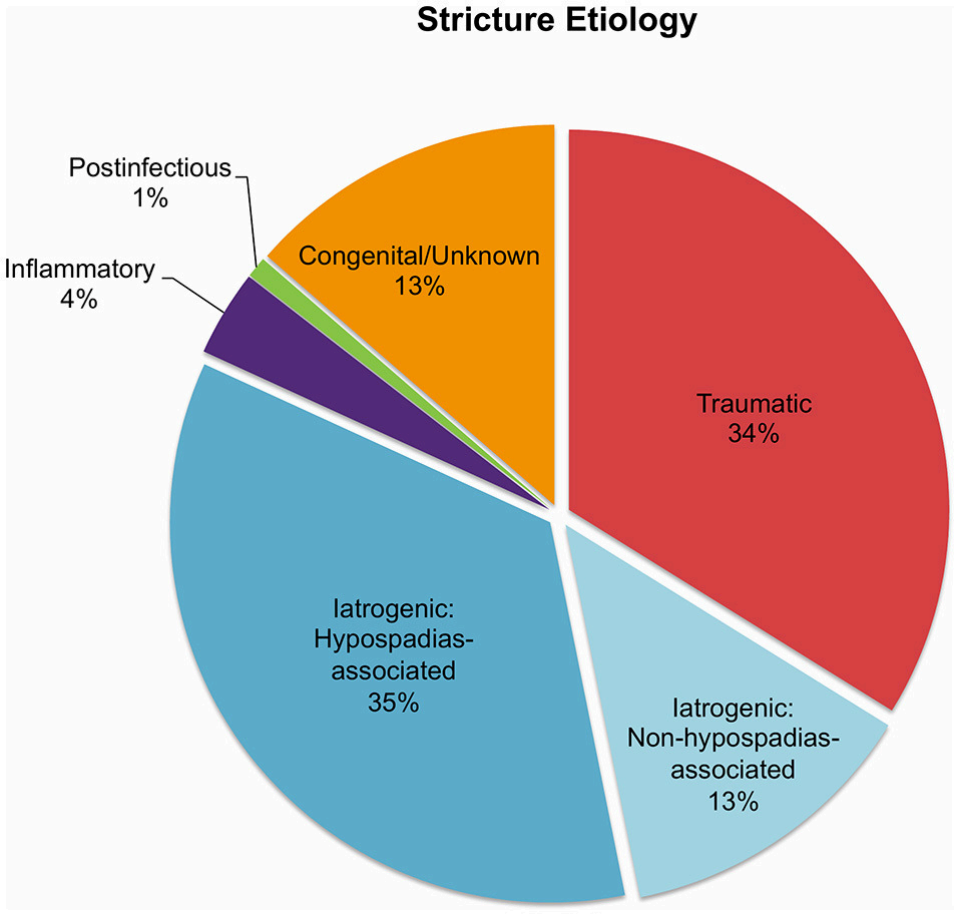
Pie charts reporting the proportions of urethral stricture etiology in all 682 patients included into the present literature review.

**Figure 2:**
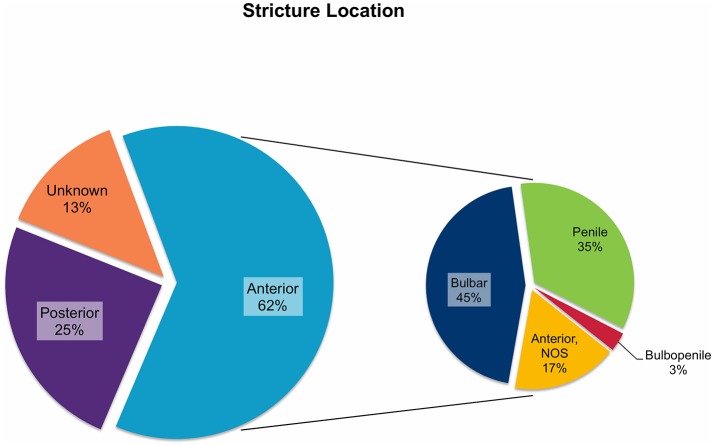
Pie charts reporting the proportions of urethral stricture location in all 682 patients included into the present literature review. NOS, not otherwise specified.

In all 682 patients, stricture etiology was mostly iatrogenic (48%), followed by traumatic (34%), and congenital (13%) strictures. Postinfectious (1%) and inflammatory strictures (4%) were rather rare (Figure 1). Given that this review aimed to focus on anterior stricture location, the majority of patients (62%) did present with an anterior urethral stricture, of which 45% had bulbar, 35% had penile, and 3% had bulbopenile strictures (Figure 2). In order not to omit data from studies reporting on outcomes in children with anterior and others with posterior strictures, we included those studies into the review. Thus, there were 25% of patients with posterior strictures and 13% in which the stricture location was not further specified (Figure 2).

## Presentation and Preoperative Evaluation

Clinical symptoms of pediatric urethral strictures are heterogeneous and the diagnosis should be ruled out if no other underlying reason can be found. Children may present with hematuria, pain, nighttime and/or daytime wetting, urinary tract infections, decreased stream, high post-void residual volume, straining to void, or dysuria (2). According to the SIU/ICUD consultation, uroflowmetry cannot be relied upon to rule out a clinically relevant stricture, and thus, combined retrograde urethrography and voiding cystourethrography as well as endoscopy are recommended as reliable diagnostic procedures (2).

## Endoscopic Treatment

Overall, seven studies reported on outcomes after endoscopic treatment (urethrotomy or dilation) (4–10) and four studies reported on comparative outcomes following endoscopic treatment vs. urethroplasty (22–25) (Tables 2, 3). Of note, only two articles performed a (partially) prospective data collection (4, 10) and thus, the level of evidence was low. The chosen definition of treatment success was heterogeneous, whereas most authors used the relatively easily assessable definition of asymptomatic voiding and no clinical symptoms (4, 5, 7, 8, 10, 23–25). Two studies did not define treatment success at all (6, 22), and seven articles chose radiographic evidence of urethral patency or no further need of any intervention as an adjunct definition of a successful surgery (4, 5, 8–10, 24, 25). The average cumulative success rate of urethrotomy or dilation in 334 patients (Tables 2, 3) was 46% (range: 21–75). Follow-up intervals varied across the studies, ranging from a median follow-up of 11.5 months (6) to an average of roughly 6.5 years (7, 9, 23, 24) Some authors included patients undergoing different treatment approaches (cold knife and laser urethrotomy or dilation) into their analyses. There were only three studies with distinguishable outcomes after dilation: overall, 23 of 58 patients (40%) recurred at a follow-up of at least 12 months (5, 22, 25). In two studies, patients undergoing urethrotomy or dilation were grouped together and thus, outcomes were not discriminable (23, 24). Thus, the lack of granularity of data and generally small sample sizes did not allow drawing any conclusion regarding a superiority of either urethrotomy (cold knife or laser) or dilation over each other. Of note, Aboulela and colleagues compared a prospectively followed cohort of 21 children undergoing holmium laser urethrotomy to a historical cohort of 21 children undergoing cold knife urethrotomy (4). Despite there was no statistically significant difference in treatment success (i.e., no voiding difficulty with improved Qmax to ≥15 mL/s confirmed by a normal urethrography) between both groups (*P* = 0.064), success rates differed clinically (67 vs. 38%). However, given the small sample size and differing follow-up (median of 12 and 24 months in the holmium laser and cold knife cohort, respectively), the authors' conclusion of the superiority of laser treatment over cold knife urethrotomy should be discussed under high scrutiny.

**Table 2:** Summary characteristics of included studies of patients who underwent endoscopic treatment (dilation or urethrotomy) for pediatric urethral stricture.

**Author**	**Year of study**	**Number of patients**	**Stricture etiology**	**Stricture location**	**Stricture length**	**Surgical approach**	**Age at surgery**	**Definition of treatment success**	**Success rate**	**Length of follow-up**	**Type of study**
Aboulela et al. (4)	2018	42	Congenital/Unknown (*N* = 23); Traumatic (*N* = 10); Iatrogenic (*N* = 9)	Bulbar/Penile (*N* = 20); Posterior (*N* = 22)	Mean: 1.0 ± 0.4 cm	Ho:YAG laser (*N* = 21; prospective cohort); Cold knife (*N* = 21; retrospective cohort)	Mean: 6.3 ± 3.2 years	No voiding difficulty with improved Qmax to ≥15 mL/s that was confirmed by a normal VCUG	Ho:YAG: 67%; Cold knife: 38%; No difference in treatment success (*P* = 0.064)	Ho:YAG: Median 12 months; Range: 6–34; Cold knife: Median 24 months; Range: 8–32; No difference in follow-up (*P* = 0.1)	Combined retrospective and prospective
Chiang et al. (5)	2007	52	Iatrogenic (*N* = 44); Congenital/Unknown (*N* = 4); Traumatic (*N* = 2); Postinfectious (*N* = 2)	Anterior (*N* = 32); Posterior (*N* = 20)	N/S	Guide wire and sheath dilator (*N* = 52)	Mean: 5.6 ± 2.3 years; Range: 2–18	No clinical symptoms or no endoscopic evidence of recurrence	39%	Mean: 4.5 ± 2.4 years; Range: 3.8–6.5	Retrospective
Faerber et al. (6)	1994	12	Iatrogenic (*N* = 10); Traumatic (*N* = 2)	Anterior (*N* = 12)	< 5 mm (*N* = 12)	Nd:YAG laser (*N* = 12)	Median: 7.5 years; Range: 2–18	N/S	75%	Median: 11.5 months; Range: 7–16	N/S
Hafez et al. (7)	2005	31	Iatrogenic (*N* = 25); Congenital/Unknown (*N* = 1); Traumatic (*N* = 5)	Bulbar (*N* = 15); Penile (*N* = 12); Posterior (*N* = 4)	< 1 cm (*N* = 15); ≥1 cm (*N* = 16)	Cold knife (*N* = 29); Ho:YAG laser (*N* = 2)	Mean: 11 years; Range: 2–18	Asymptomatic voiding without clinical evidence of residual stricture (good flow rate and absence of residual urine)	36%	Mean: 6.6 years; Range: 2–20	Retrospective
Hsiao et al. (8)	2003	50	Iatrogenic (*N* = 34), Congenital/Unknown (*N* = 11), Traumatic (*N* = 5)	Bulbar (*N* = 31); Penile (*N* = 12); Posterior (*N* = 6); Unknown (*N* = 1)	N/S	Cold knife (*N* = 45); Electrocautery loop (*N* = 4); Ho:YAG laser (*N* = 1)	Mean: 7.7 years; Range: 6–17	No symptoms for >12 months and no subsequent procedure required	50%	Median: 2 years; Range: 1–7 (*N* = 40)	Retrospective
Launonen et al. (9)	2014	34	Iatrogenic (*N* = 23); Traumatic (*N* = 3); Congenital/Unknown (*N* = 8)	Bulbar (*N* = 25); Penile (*N* = 7); Bulbopenile (*N* = 2)	>2 cm (*N* = 5); ≤ 2 cm (*N* = 29)	Cold knife (*N* = 34)	Median: 6.3 years; Range: 0.2–16.3	No subsequent procedure required	26%	Median: 6.6 years; Range: 0.6–17.4	Retrospective
Shoukry et al. (10)	2016	29	Iatrogenic (*N* = 9); Traumatic (*N* = 6); Congenital/Unknown (*N* = 14)	Bulbar (*N* = 12); Penile (*N* = 3); Posterior (*N* = 14)	0.5–0.9 cm (*N* = 16); 1–2 cm (*N* = 13)	Ho:YAG laser (*N* = 29)	Median: 5.0 years; Range: 2–13	No postoperative voiding difficulty, postoperative maximum flow rate (Qmax) >15 mL/s and normal urethrography	62%	Range: 12–15 months	Prospective

*Ho:YAG, holmium yttrium aluminium garnet; N/S, not specified; VCUG, voiding cystourethrography*.

**Table 3:** Summary characteristics of included studies on comparative outcomes after both endoscopic and open surgical treatment for pediatric urethral stricture.

**Author**	**Year of study**	**Number of patients**	**Stricture etiology**	**Stricture location**	**Stricture length**	**Surgical approach**	**Age at surgery**	**Definition of treatment success**	**Success rate**	**Length of follow-up**	**Type of study**
Gobbi et al. (25)	2017	7	Congenital/ Unknown (*N* = 7)	Bulbar (*N* = 3); Penile (*N* = 4)	>1 cm (*N* = 4); < 1cm (*N* = 3)	Urethrotomy (*N* = 2); Urethroplasty (*N* = 2); Dilation (*N* = 3)	Mean: 3.7 months ± 3.5; Range: 1–10	Absence of urinary symptoms at follow-up, radiologic or endoscopic documentation of resolution of the stricture	Urethrotomy: 50%; Urethroplasty: 100%; Dilation: 67%	>12 months; not further specified	Retrospective
Banks et al. (22)	2009	12	Congenital/ Unknown (*N* = 12)	Bulbar (*N* = 10); Posterior (*N* = 2)	N/S	Urethrotomy: Cold knife (*N* = 8); Dilation (*N* = 3); Urethroplasty: EPA (*N* = 1)	< 1 year (*N* = 6); Median: 14 years; Range: 3–15 (*N* = 6)	N/S	Urethrotomy: 38%; Dilation: 33%; Urethroplasty: 100%	Mean: 19 months; Range: 3–84	Retrospective
Duel et al. (23)	1998	38	Iatrogenic (*N* = 38)	N/S	N/S	Urethrotomy/ Dilation (*N* = 29); Urethroplasty [Duplay tube (*N* = 3); Pedicled onlay flap (*N* = 4); Bladder mucosa tube (*N* = 1)]; Lost to follow-up (*N* = 1)	Mean at initial hypospadias repair: 2 years; Range: 0.6–5.8; Mean interval from hypospadias repair to stricture: 27 months; Range 1–150	Absence of urinary symptoms	Urethrotomy/ Dilation: 21%; Urethroplasty: 88%	Mean: 6.3 years; Range: 0.1–13	Retrospective
Gargollo et al. (24)	2011	88	Iatrogenic (*N* = 88)	Distal (*N* = 46); Mid-shaft (*N* = 11); Proximal (*N* = 31)	Mean: 1.7 ± 0.7 cm (*N* = 37)	Dilation/ Urethrotomy (*N* = 39); Urethroplasty: technique N/S (*N* = 49)	Dilation/ Urethrotomy—Mean: 64.6 ± 47.5 months; Urethroplasty—Mean: 57.1 ± 49.1 months	Absence of symptoms for >12 months and no subsequent procedure required	Dilation/ Urethrotomy: 38%; Urethroplasty: 53%; Overall: 47%	Dilation/ Urethrotomy—Mean: 73.8 ± 49.9 months; Urethroplasty—Mean: 81.0 ± 63.1 months	Retrospective

*EPA, excision and primary anastomosis; N/S, not specified*.

## Urethroplasty

**Figure 3:**
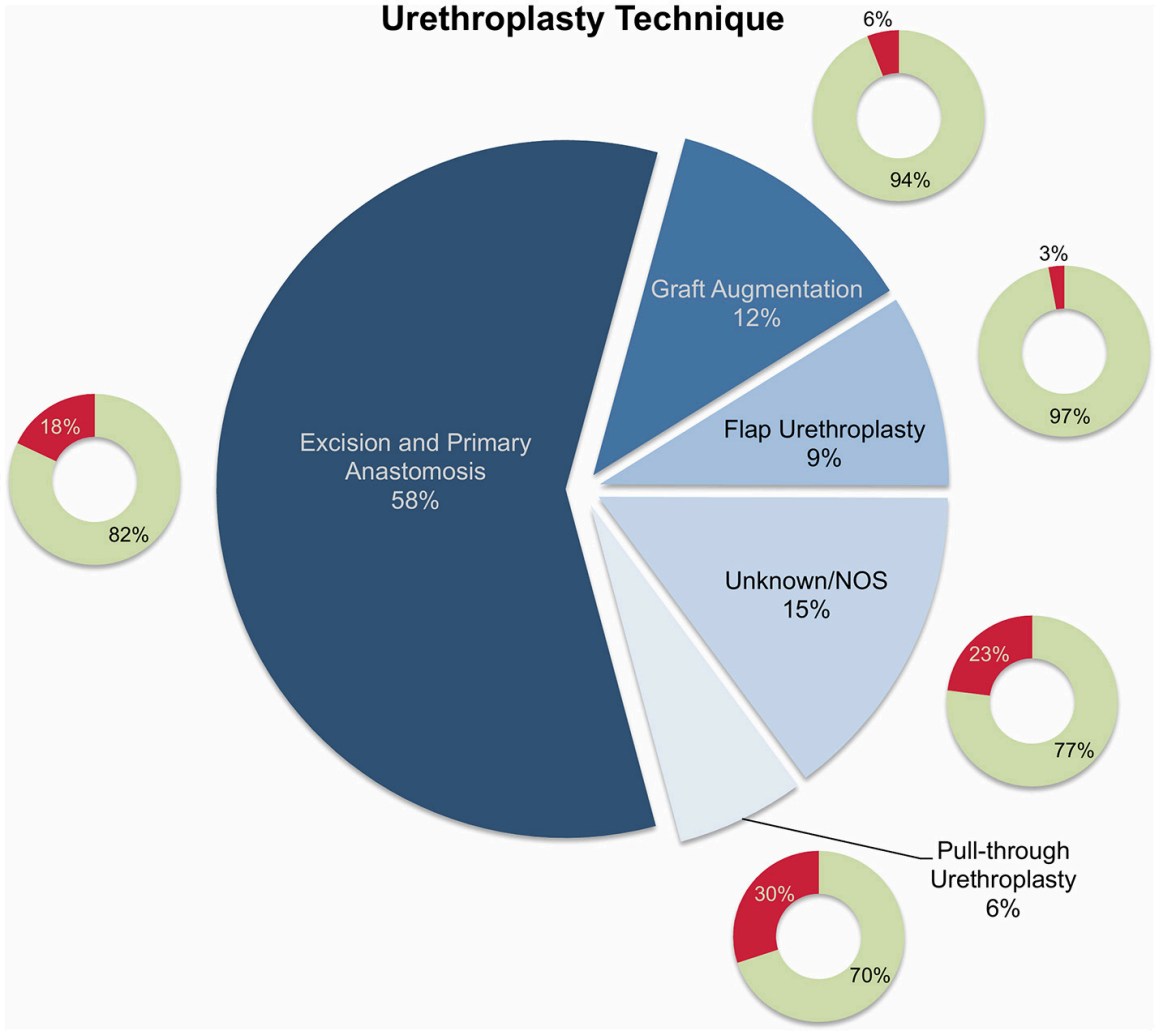
Pie chart reporting the proportions of different reconstructive techniques used in 347 patients who underwent urethroplasty and were included into the present literature review. The smaller doughnut charts represent the treatment success rates for each technique (green: success; red: failure). NOS, not otherwise specified.

Overall, 11 studies reported on outcomes after urethroplasty (11–21) and the aforementioned four studies reported on both endoscopic treatment and open surgery (22–25) (Tables 1, 3). All studies were performed retrospectively without exception. Similar to the articles on endoscopic treatments, the definitions of (surgical) treatment success were heterogeneous. In eight of the 15 studies, the authors used relatively objective criteria such as radiographic evidence of urethral patency (11, 13, 19, 21, 25) or no further need of any intervention (11, 15, 17, 24) as a surrogate for success, whereas treatment success was determined rather clinically (absence of urinary symptoms, improvement in uroflowmetry, patient satisfaction, absence of post-void residual urine) in five (12, 14, 16, 18, 23) or was not defined in two studies (20, 22). Roughly 58% of boys undergoing urethroplasty were treated by excision and primary anastomosis, followed by graft augmentation in 12%, flap urethroplasty in 9%, and pull-through urethroplasty in 6%. The reconstructive technique was not specified in 15% of patients (Figure 3). Follow-up intervals varied across the studies, ranging from a median follow-up of 0.7 years (21) to an average of roughly 6.8 years (24). After stratifying patients according to urethroplasty technique, average cumulative success rates were 82% (range: 25–100) for excision and primary anastomosis in 206 boys, 94% (range: 75–100) for graft augmentation in 40 boys, 97% (range: 87–100) for flap urethroplasty in 30 boys, 70% for pull-through urethroplasty in 20 boys (one study), and 77% in 51 boys in whom urethroplasty technique was not further specified (Figure 3). The overall cumulative success rate of urethroplasty in 347 patients (one was lost to follow-up) was 84% (range: 25–100), irrespective of the reconstructive technique used (Tables 1, 3, Figure 3).

Given that nine of the 15 studies evaluating the role of urethroplasty in children with urethral stricture included both posterior and anterior strictures (14–22), the reporting of outcomes for isolated anterior strictures was hardly possible. Several other pitfalls have to be considered when interpreting the present data and drawing therapeutical conclusions. Overall, the evidence on which this review was based on was very low with almost all studies being invariably of retrospective nature. In addition, the reported outcomes in the studies included into this review lack homogeneity, as the definition of treatment success was chosen at the discretion of each author—an issue which is seen quite often in reconstructive urology and it is still an ongoing debate on how to define success after reconstructive urological surgery (27, 28). Furthermore, a detailed assessment of graft placement techniques such as ventral, dorsal or lateral placement and differences in outcomes was not possible given the lack of granularity within the primary data.

Generally, several stricture characteristics have to be considered when opting for a reconstructive technique. Length, location, etiology, and previous interventions may hereby guide in treatment decisions. The majority of the children were treated by excision and primary anastomosis, a feasible option in short posterior or bulbar strictures < 1 cm. Considering the studies included into this review, success rates after graft augmentation or flap urethroplasty were markedly higher compared to those in patients who underwent excision and primary anastomosis (roughly 95 vs. 82%, respectively; Figure 3). The aforementioned limitations of the present data left aside, one should always keep in mind the anatomical differences in children as opposed to adults when favoring one technique over another. The urethral caliber is smaller, the tissue more delicate and less elastic, and many children have a history of multiple surgical interventions, specifically in case of iatrogenic hypospadias-associated urethral strictures. Hypospadias repair is the leading cause of subsequent urethral stricture formation and this is somehow reflected by roughly 50% of the strictures in patients included into this review were caused iatrogenically. Of note, roughly three-fourths of these patients had a history of previous hypospadias surgery (Figure 1). Given that pronounced scar formation and lack of sufficient sponge tissue hamper an adequate reconstruction significantly, some authors have suggested additive maneuvers to improve vascular supply and tissue coverage. Scarred tissue is commonly attached firmly to the cavernous bodies, which complicates urethral mobilization. On the other hand, ventral placement of the graft lacks the support of the corporeal bodies, which may promote formation of diverticula and sacculation. Djordjevic et al. introduced a combined technique of a ventral only buccal mucosal graft with an anchoring of the graft to the surrounding periurethral tissue to prevent folding and retraction. In 15 boys with urethral stricture following failed hypospadias surgery, the success rate was 100% after a mean follow-up of 37 months, and only one boy underwent a minor fistula repair (13).

Impressive data with regard to comparative outcomes in stricture treatment following hypospadias repair have been presented by Duel et al. (23) The authors compared eight children who underwent upfront urethroplasty to 29 children who underwent urethrotomy and found that those undergoing open surgery fared significantly better (88 vs. 21% success at a mean follow-up of 6.3 years). Similar but somehow less pronounced findings were made by Gargollo et al. (24) who also demonstrated a clinically relevant superiority of initial urethroplasty over endoscopic treatment (53 vs. 38% success at a follow-up of roughly 6 years; *P* > 0.05) in patients with iatrogenic stricture after hypospadias repair. Interestingly, the authors evaluated secondary success rates after another procedure in case of stricture recurrence and found that success rates ranged between roughly 60–70% whenever urethroplasty was performed at any time during follow-up, whereas repeat urethrotomy was successful in only 17% of patients (24). Whereas, the case series by Gobbi et al. (25) and Banks et al. (22) take the same line for congenital strictures, patient samples are too small to draw reliable conclusions.

A clear distinction between inflammatory and postinfectious strictures in children is commonly not feasible. There is some evidence suggesting an inflammatory etiology of stricture formation caused by lichen sclerosus (29), several autoimmune disorders (26), bulbar urethritis, and urethrorrhagia (30). Postinfectious strictures are mainly caused by recurrent gonococcal urethritis (26). Inflammatory strictures caused by lichen sclerosus are rare in children (Figure 1), but nevertheless pose a significant challenge to the reconstructive surgeon. Generally, repeat procedures such as multiple urethrotomies should be avoided and the lichenoid tissue should be dissected in order to avoid excessive scar formation and mitigate the need of further treatment. Currently, the use of genital skin as a tissue flap is not considered appropriate, given that it remains prone to the same disease process (31). Thus, promising outcomes using non-skin, oral mucosal grafts have been reported in the adult literature (32, 33), and Ashraf et al. recently reported on five boys who underwent one-stage Asopa buccal mucosal graft urethroplasty for lichen sclerosus-associated penile urethral stricture with a median length of 2.7 cm. There was no treatment failure at a median follow-up of roughly 3 years (12).

## Conclusions

Although there are several surgical options at hand for pediatric urethral stricture, the paucity of literature, which is mainly based on small monocentric series, often hampers treatment decisions for this rare disease, specifically when opting for therapeutical sequences, and gauging different strategies. However, there is some evidence available and when meticulously summarizing individual patient level data, the therapeutical perspective may be broadened, even if high-level evidence from multi-institutional, prospective collaborations with sound statistical methods are currently lacking. Pediatric anterior urethral strictures are mostly iatrogenically caused of which the majority of cases are hypospadias-associated, followed by traumatic, and congenital strictures. Endoscopic strategies such as dilation and direct vision internal cold knife or laser urethrotomy are ineffective in the long-term and should not be chosen as a first-line treatment. Urethroplasty should be preferred as definitive therapy to avoid multiple interventions and diminish clinical visits and a long time of suffering. Excision and primary anastomosis may be preferred if the stricture is short and mobilization is anatomically feasible with reported cumulative success rates of roughly 82%. Graft augmentation or flap urethroplasty did perform somewhat better, with a cumulative success rate of roughly 95% when performed in referral centers in capable hands. However, sample sizes are small and thus, results should be interpreted with caution and no unequivocal recommendation can be made to favor one open surgical approach over the other. More importantly, one highly relevant outcome measure is commonly missing in the literature, as there is virtually no data on functional outcomes such as erectile and sexual function, urinary continence, and body image or cosmetic results following surgery for pediatric urethral stricture. This comes along with short follow-up periods and inconsistency and heterogeneity in outcome definitions. Thus, researchers are challenged to establish retrospective and prospective multi-institutional collaborations, incorporating granular patient level data with adequately defined outcome measures to advance our knowledge in this relatively unattended field of reconstructive urology.

## Author Contributions

MV: primary responsibility for communication with the journal during the manuscript submission, literature review, drafting the work, final approval of the version to be published, agreement to be accountable for all aspects of the work. LW: literature review, critical revision of the article, final approval of the version to be published, agreement to be accountable for all aspects of the work. SR: critical revision of the article, final approval of the version to be published, agreement to be accountable for all aspects of the work. MF: supervision, critical revision of the article, final approval of the version to be published, agreement to be accountable for all aspects of the work.

### Conflict of Interest Statement

The authors declare that the research was conducted in the absence of any commercial or financial relationships that could be construed as a potential conflict of interest.

## References

[1] Kaplan GW. Urethral strictures in children. Curr Opin Urol. (2012) 22:462–6. 10.1097/MOU.0b013e328357bc78 22918037

[2] Kaplan GW Brock JW Fisch M Koraitim MM Snyder HM. SIU/ICUD consultation on urethral strictures: urethral strictures in children. Urology (2014) 83(Suppl. 3):S71–3. 10.1016/j.urology.2013.09.010 24231203

[3] Lazzeri M Sansalone S Guazzoni G Barbagli G Incidence, causes, and complications of urethral stricture disease. Eur Urol Suppl. (2016) 15:2–6. 10.1016/j.eursup.2015.10.002

[4] Aboulela W ElSheemy MS Shoukry M Shouman AM Shoukry AI Ghoneima W. Visual internal urethrotomy for management of urethral strictures in boys: a comparison of short-term outcome of holmium laser versus cold knife. Int Urol Nephrol. (2018) 50:605–9. 10.1007/s11255-018-1809-x 29397549

[5] Chiang DT Dewan AP. Guide wire-assisted urethral dilation in pediatric urology: experience of a single surgeon. Urol J. (2007) 4:226–9. 10.22037/uj.v4i4.102 18270947

[6] Faerber GJ Park JM Bloom DA. Treatment of pediatric urethral stricture disease with the neodymium:yttrium-aluminum-garnet laser. Urology (1994) 44:264–7. 10.1016/S0090-4295(94)80146-0 8048204

[7] Hafez AT El-Assmy A Dawaba MS Sarhan O Bazeed M. Long-term outcome of visual internal urethrotomy for the management of pediatric urethral strictures. J Urol. (2005) 173:595–7. 10.1097/01.ju.0000151339.42841.6e 15643267

[8] Hsiao KC Baez-Trinidad L Lendvay T Smith EA Broecker B Scherz H. Direct vision internal urethrotomy for the treatment of pediatric urethral strictures: analysis of 50 patients. J Urol. (2003) 170:952–5. 10.1097/01.ju.0000082321.98172.4e 12913749

[9] Launonen E Sairanen J Ruutu M Taskinen S. Role of visual internal urethrotomy in pediatric urethral strictures. J Pediatr Urol. (2014) 10:545–9. 10.1016/j.jpurol.2013.11.018 24388665

[10] Shoukry AI Abouela WN ElSheemy MS Shouman AM Daw K Hussein AA. Use of holmium laser for urethral strictures in pediatrics: a prospective study. J Pediatr Urol. (2016) 12:42.e1–6. 10.1016/j.jpurol.2015.06.016 26302829

[11] Aldaqadossi HA Shaker H Kotb Y Youssof H Elgamal S. Penile fasciocutaneous flap urethroplasty for the reconstruction of pediatric long anterior urethral stricture. J Pediatr Urol. (2018) 14:555.e1–6. 10.1016/j.jpurol.2018.07.008 30131215

[12] Ashraf J Turner A Subramaniam R. Single-stage urethroplasty with buccal mucosal inlay graft for stricture caused by balanitis xerotica obliterans in boys: outcomes in the medium term. J Pediatr Urol. (2018) 14:66.e1–5. 10.1016/j.jpurol.2017.09.015 29150196

[13] Djordjevic ML Kojovic V Bizic M Majstorovic M Vukadinovic V Korac G. “Hanging” of the buccal mucosal graft for urethral stricture repair after failed hypospadias. J Urol. (2011) 185(Suppl. 6):2479–82. 10.1016/j.juro.2011.01.036 21527203

[14] Hafez AT El-Assmy A Sarhan O El-Hefnawy AS Ghoneim MA. Perineal anastomotic urethroplasty for managing post-traumatic urethral strictures in children: the long-term outcome. BJU Int. (2005) 95:403–6. 10.1111/j.1464-410X.2005.05309.x 15679803

[15] Pfalzgraf D Isbarn H Meyer-Moldenhauer WH Fisch M Riechardt S. Etiology and outcome of the perineal repair of posterior and bulbar urethral strictures in children: a single surgeon experience. J Pediatr Urol. (2013) 9(6 Pt A):769–74. 10.1016/j.jpurol.2012.09.007 23073040

[16] Rourke KF McCammon KA Sumfest JM Jordan GH Kaplan GW. Open reconstruction of pediatric and adolescent urethral strictures: long-term followup. J Urol. (2003) 169:1818–21; discussion: 21. 10.1097/01.ju.0000056035.37591.9f 12686852

[17] Shenfeld OZ Gdor J Katz R Gofrit ON Pode D Landau EH. Urethroplasty, by perineal approach, for bulbar and membranous urethral strictures in children and adolescents. Urology (2008) 71:430–3; discussion: 3–4. 10.1016/j.urology.2007.09.072 18342179

[18] Sunay M Karabulut A Dadali M Bagbanci S Emir L Erol D. Single-institution outcomes of open reconstruction techniques for management of pediatric and adolescent post-traumatic urethral strictures. Urology (2011) 77:706–10. 10.1016/j.urology.2010.07.476 20970838

[19] Trachta J Moravek J Kriz J Padr R Skaba R. Pediatric bulbar and posterior urethral injuries: operative outcomes and long-term follow-up. Eur J Pediatr Surg. (2016) 26:86–90. 10.1055/s-0035-1566102 26540444

[20] Vashishtha S Sureka SK Kumar J Prabhakaran S Kapoor R Ansari MS. Predictors for recurrence after urethroplasty in pediatric and adolescent stricture urethra. J Pediatr Urol. (2014) 10:268–73. 10.1016/j.jpurol.2013.08.014 24726239

[21] Voelzke BB Breyer BN McAninch JW. Blunt pediatric anterior and posterior urethral trauma: 32-year experience and outcomes. J Pediatr Urol. (2012) 8:258–63. 10.1016/j.jpurol.2011.05.010 21664873 PMC3565598

[22] Banks FC Griffin SJ Steinbrecher HA Malone PS. Aetiology and treatment of symptomatic idiopathic urethral strictures in children. J Pediatr Urol. (2009) 5:215–8; discussion 9–20. 10.1016/j.jpurol.2009.01.004 19230775

[23] Duel BP Barthold JS Gonzalez R. Management of urethral strictures after hypospadias repair. J Urol. (1998) 160:170–1. 10.1016/S0022-5347(01)63083-0 9628643

[24] Gargollo PC Cai AW Borer JG Retik AB Management of recurrent urethral strictures after hypospadias repair: is there a role for repeat dilation or endoscopic incision? J Pediatr Urol. (2011) 7:34–8. 10.1016/j.jpurol.2010.03.007 20462798

[25] Gobbi D Fascetti Leon F Gnech M Midrio P Gamba P Castagnetti M. Management of congenital urethral strictures in infants. Case series. Urol J. (2018). 10.22037/uj.v0i0.4045. [Epub ahead of print]. 30058064

[26] Latini JM McAninch JW Brandes SB Chung JY Rosenstein D. SIU/ICUD consultation on urethral strictures: epidemiology, etiology, anatomy, and nomenclature of urethral stenoses, strictures, and pelvic fracture urethral disruption injuries. Urology (2014) 83(Suppl. 3):S1–7. 10.1016/j.urology.2013.09.009 24210733

[27] Meeks JJ Erickson BA Granieri MA Gonzalez CM. Stricture recurrence after urethroplasty: a systematic review. J Urol. (2009) 182:1266–70. 10.1016/j.juro.2009.06.027 19683309

[28] Angermeier KW Rourke KF Dubey D Forsyth RJ Gonzalez CM. SIU/ICUD consultation on urethral strictures: evaluation and follow-up. Urology (2014) 83(Suppl. 3):S8–17. 10.1016/j.urology.2013.09.011 24275285

[29] Pugliese JM Morey AF Peterson AC. Lichen sclerosus: review of the literature and current recommendations for management. J Urol. (2007) 178:2268–76. 10.1016/j.juro.2007.08.024 17936829

[30] Walker BR Ellison ED Snow BW Cartwright PC. The natural history of idiopathic urethrorrhagia in boys. J Urol. (2001) 166:231–2. 10.1016/S0022-5347(05)66132-0 11435875

[31] Stewart L McCammon K Metro M Virasoro R. SIU/ICUD consultation on urethral strictures: anterior urethra-lichen sclerosus. Urology (2014) 83(Suppl. 3):S27–30. 10.1016/j.urology.2013.09.013 24268357

[32] Xu YM Feng C Sa YL Fu Q Zhang J Xie H. Outcome of 1-stage urethroplasty using oral mucosal grafts for the treatment of urethral strictures associated with genital lichen sclerosus. Urology (2014) 83:232–6. 10.1016/j.urology.2013.08.035 24200196

[33] Kulkarni S Barbagli G Kirpekar D Mirri F Lazzeri M. Lichen sclerosus of the male genitalia and urethra: surgical options and results in a multicenter international experience with 215 patients. Eur Urol. (2009) 55:945–54. 10.1016/j.eururo.2008.07.046 18691809

